# Cancer-Associated Venous Thromboembolism: Changes Over the Past 20 Years∗

**DOI:** 10.1016/j.jaccao.2023.10.007

**Published:** 2023-12-19

**Authors:** Wei Xiong, Ryuki Chatani, Yugo Yamashita

**Affiliations:** a Department of Cardiovascular Medicine, Graduate School of Medicine, Kyoto University, Kyoto, Japan; b Department of Pulmonary and Critical Care Medicine, Xinhua Hospital, Shanghai Jiaotong University School of Medicine, Shanghai, China; c Department of Cardiovascular Medicine, Kurashiki Central Hospital, Kurashiki, Japan

**Keywords:** epidemiology, peripheral vascular disease, thrombosis

**Figure:**
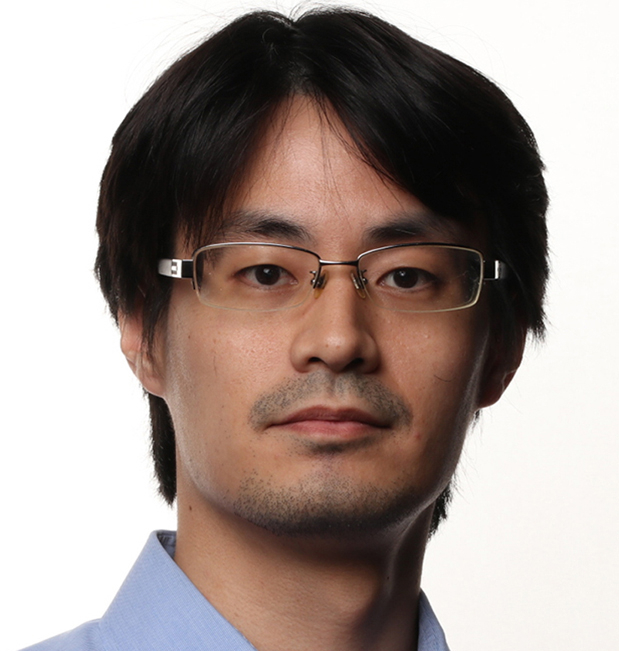

Cancer-associated thrombosis that comprises venous thromboembolism (VTE) and arterial thromboembolism is one of the major cardiovascular complications during cancer treatment and is a leading cause of death after cancer-related death in patients with cancer.^1^ VTE, including deep venous thrombosis and pulmonary embolism, occurs at a rate in cancer patients about 4- to 7-fold higher than in healthy individuals.^1^ In addition, the recurrence of VTE is more common in patients with cancer than those without. Although the thrombotic risk is high in patients with cancer, the risk of bleeding is also higher in patients with cancer than those without. Thus, patients with cancer-associated VTE are a challenging population when considering decisions such as optimal anticoagulation strategies, which is a significant issue in the field of cardio-oncology.^2^, ^3^, ^4^, ^5^

The life expectancy of patients with cancer has greatly increased over several decades with the rapid improvements in anticancer therapy.^6^ In addition to prolonged life expectancy, increased use of thrombogenic anticancer treatments, extensive use of central venous catheterization, and a better awareness of cancer-associated thrombosis among clinicians might lead to an increasing incidence of cancer-associated VTE.^7^, ^8^, ^9^, ^10^ Alongside this, the management strategies of prophylaxis and treatment for cancer-associated VTE have been updated in several guidelines,^2^, ^3^, ^4^, ^5^ which might also impact the clinical outcomes of cancer-associated VTE. However, clinicians encounter a variety of patients with cancer-associated VTE in daily clinical practice, and the established recommendations in the guidelines might not always be followed. Despite the established evidence from randomized controlled trials demonstrating the usefulness of long-term low molecular weight heparin over warfarin for cancer-associated VTE, previous studies reported that only half of patients received long-term low molecular weight heparin according to the recommendations.^11^^,^^12^ Thus, it is important to understand the current epidemiology of cancer-associated VTE using “real-world” evidence, which may be helpful to understand the unsolved issues and unmet needs in the current treatment era. Although there have been several studies of these issues,^8^, ^9^, ^10^ detailed data on temporal changes in management strategies and clinical outcomes for cancer-associated VTE have been scarce.

In this issue of *JACC: CardioOncology*, Bertoletti et al^13^ comprehensively reported the trends in clinical features, treatment, and outcomes for cancer-associated VTE from 2001 to 2020 from the Registro Informatizado de la EnfermedadTromboEmbólica database, which is an internationally recognized registry. The current study is a large-scale and detailed study investigating the temporal trends in management strategies and clinical outcomes. The prevalence of patients presenting with pulmonary embolus increased over time from 44% in 2001 to 2005 to 55% in 2016 to 2020, consistent with previous reports.^8^^,^^9^ The 30-day mortality rate decreased from 11.9% to 8.4%, which is also consistent with a previous report from 2006 to 2017.^8^ As for long-term anticoagulation strategies, the use of direct oral anticoagulant (DOAC) increased after 2016, although the proportions of DOACs was still relatively low at 12.3% between 2016 and 2020 in the current study. The 30-day symptomatic VTE recurrence rate decreased from 3.1% in 2001 to 2005 to 1.1% in 2016 to 2020, and the 30-day major bleeding rate declined from 3.1% to 2.2%. A previous study reported that DOACs could confer a reduced risk of recurrent VTE without an increase of major bleeding despite a potential increased risk of clinically relevant nonmajor bleeding.^3^ The current study revealed that the temporal change of major bleeding rates was declining, possibly from changes in treatment patterns. Notably, the 30-day major bleeding rate was 2-fold higher than the 30-day VTE recurrence rate in 2016 to 2020, although both adverse outcomes were declining. In the Hokusai-VTE trial, the 30-day major bleeding rate was 7.6%, whereas the 30-day VTE recurrence rate was 0.8%.^14^ These results impress upon us the concerns with bleeding complications in the current era.

From a practical point of view, the real-world evidence of cancer-associated VTE is important, and considering the current results, clinical outcomes for cancer-associated VTE have improved over the past 2 decades, likely in part through the advances in the management strategies for cancer-associated VTE. The current study by Bertoletti et al^13^ provides useful information about unresolved and clinically relevant issues in the VTE and cardio-oncology field. In terms of future directions, additional studies are warranted to confirm these current results. In addition, several issues that were not investigated in the current study including long-term outcomes beyond 30 days should be further investigated. Overall, the authors should be congratulated for reporting these clinically important results from the RIETE database.

## Funding Support and Author Disclosures

Dr Yamashita has received lecture fees from Bayer Healthcare, Bristol-Myers Squibb, Pfizer, and Daiichi Sankyo; and has received grant support from Bayer Healthcare and Daiichi Sankyo. All other authors have reported that they have no relationships relevant to the contents of this paper to disclose.
